# Vertebrate Responses against Arthropod Salivary Proteins and Their Therapeutic Potential

**DOI:** 10.3390/vaccines9040347

**Published:** 2021-04-05

**Authors:** Olayinka Olajiga, Andrés F. Holguin-Rocha, Meagan Rippee-Brooks, Megan Eppler, Shanice L. Harris, Berlin Londono-Renteria

**Affiliations:** 1 Vector Biology Laboratory, Department of Entomology, Kansas State University, Manhattan, KS 66506, USA; olayinkajiga@ksu.edu (O.O.); aholguin@ksu.edu (A.F.H.-R.); megeppler96@ksu.edu (M.E.); harrissl@vet.k-state.edu (S.L.H.); 2 Department of Biology, Missouri State University, Springfield, MO 65897, USA; mrippeebrooks@gmail.com

**Keywords:** vector-borne, salivary proteins, arbovirus, vaccines

## Abstract

The saliva of hematophagous arthropods contains a group of active proteins to counteract host responses against injury and to facilitate the success of a bloodmeal. These salivary proteins have significant impacts on modulating pathogen transmission, immunogenicity expression, the establishment of infection, and even disease severity. Recent studies have shown that several salivary proteins are immunogenic and antibodies against them may block infection, thereby suggesting potential vaccine candidates. Here, we discuss the most relevant salivary proteins currently studied for their therapeutic potential as vaccine candidates or to control the transmission of human vector-borne pathogens and immune responses against different arthropod salivary proteins.

## 1. Introduction

Vector-borne diseases account for more than 17% of all infectious diseases globally, with dengue fever via dengue virus (DENV) as the most prevalent arthropod-borne virus or arbovirus [1,2]. The main control strategies to decrease vector-borne diseases are based on vector control, which aims to prevent or decrease exposure to infective bites. This strategy includes the use of a wide range of tools from personal protective equipment, physical devices (i.e., bed nets), and insecticides [3,4] to insecticide/larvicide use [5]. However, the sustainability of vector control is an issue and previous studies suggest that this intervention alone might be insufficient to decrease the annual burden [6,7,8]. Alternative disease control methods such as vaccines and specific therapies are urgently needed.

**Figure 1:**
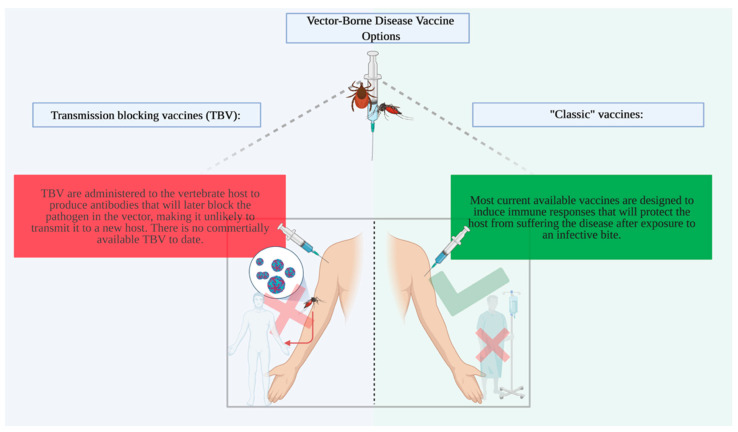
Vaccine options to prevent vector-borne disease transmission. Transmission blocking vaccines (TBVs) are administered to the vertebrate host to induce immune responses that will later block pathogen development in the arthropod vector during or after the blood feeding. “Classic” vaccines are designed to prevent vertebrate infection and disease after exposure to an “infective” bite (figure created with BioRender.com) (accessed on 4 March 2021).

Vaccines represent a viable alternative to protect the public against infection, and in the case of vector-borne diseases, there are several options commercially available. We call these vaccines “classic vaccines” because they protect the vertebrate host from getting the disease. A second group of vaccines is called transmission-blocking vaccines (TBVs) (reviewed in detail by Bakhshi et al., 2018, and Londono-Renteria et al., 2016) [9,10]. The objective of TBVs is to prevent the survival of pathogens in the arthropod host by “blocking” transmission (Figure 1). Currently, there are no commercially available TBVs (Table 1).

However, several vaccines are currently available to prevent disease in humans. At least two vaccines against yellow fever (YFV) have been proved safe for human use since 1938 they are described in Table 1 [11]. Also, a vaccine against DENV, Dengvaxia, was licensed in 2016 in select countries, but controversies surrounding the phenomenon of possible vaccine-induced immune potentiation of more severe illness in children have halted their use in naïve populations [12]. Vaccines against Japanese encephalitis virus (JEV) were developed in the 1930s and are currently available as a live attenuated vaccine, the SA14–14–2 JE vaccine, which was approved by the WHO for use in national immunization programs in Asia and IXIARO, and has also been licensed since 2009 for use in several countries including the US and Canada [13,14]. However, there are a significant number of vector-borne diseases without vaccine options and substantial research efforts are currently focused on identifying suitable targets to expand the number of vector-borne diseases that could be controlled through vaccination campaigns [15,16].

**Table 1:** List of current licensed vaccines against arboviruses.

Pathogen	Vaccine Name	Year Licensed	Efficacy	Component	References
Dengue virus	Dengvaxia (CYT-TDV)	2015	25–59%	Live attenuated tetravalent chimeric vaccine	[17]
Yellow fever virus	YF-VAX	2001	>95%	Live attenuated yellow fever virus strain 17D-204	[11,18]
Yellow fever virus	STAMARIL	1986	Comparable to YF-VAX	Live attenuated yellow fever virus strain 17D-204	[19]
Japanese Encephalitis virus	IXIARO/JESPECT	2009	99.3%	Live attenuated SA-14-14-2	[13,20]

Current vaccines contain “pathogen-derived” molecules and are designed to induce protective immunity by targeting pathogen invasion and survival mechanisms, thereby blocking their replication in the host. However, most pathogens transmitted by arthropods are deposited under the vertebrate skin along with the arthropod saliva during blood feeding [21]. Saliva is composed of a wide range of molecules whose objective is to counteract the vertebrate hemostasis and facilitate blood uptake [22]. Salivary proteins can be cataloged into three main groups—anticoagulants, vasodilators, and immunoregulators—but some proteins may have redundant functions, all to increase the chances of successful meal acquisition [23]. Recently, there has been an increasing interest in different options for vaccines against vector-borne diseases. These options include identifying salivary proteins associated with potentiating pathogen survival and disease progression. These vaccines will contain “mosquito-derived” molecules.

Compelling evidence suggests that arthropod salivary proteins induce profound changes in immune responses in the vertebrate host both locally (at the feeding site) and systemically [24], eventually providing a vehicle for pathogen transmission [25,26,27], and pathogens usually take advantage of the immunomodulatory properties of the vector saliva to successfully establish infection [22,28]. These salivary proteins also induce significant humoral and cellular immune responses in the host [24,29]. There is evidence that protective immunity against vector-borne diseases may not only be directed against the pathogen, but also against the vector salivary components [30,31]. Thus, an increased number of studies are focused on identifying key salivary proteins from the main vectors of human disease and characterizing their potential as vaccine candidates (Table 2). This study focused on the recent arthropod salivary proteins that have been identified as potential vaccine candidates for humans to prevent arboviruses transmitted among humans by *Aedes aegypti* mosquitoes and other pathogens transmitted by ticks.

## 2. Arthropod Salivary Protein Candidates for Vaccines

Since the presence of mosquito saliva in the skin has profound effects on pathogen replication and immunomodulation, leading to disease progression [29,36], several salivary proteins have been characterized as potential vaccine candidates [41]. The rationale is that blocking the enhancing effect of such salivary proteins may block infection. In this regard, several proteins are currently being studied for their potential to block infection in the vertebrate host.

The *Ae. aegypti* Bacteria-Responsive protein 1 (AgBR1) is identified using serum from mice chronically exposed to *Ae. aegypti* bites and is associated with inflammation and neutrophil recruitment in the skin following a blood meal [34]. Recent studies demonstrate that neutrophil recruitment is key in the initiation of a cascade of events leading to the recruitment of virus target cells [27]. Interestingly, AgBR1 antiserum decreases inflammation in the skin, and antibodies against this protein suppress viral dissemination and induce protection against the lethal Zika virus (ZIKV) infection [34,42]. They also reduce the initial viral load of West Nile virus (WNV) following exposure to an infectious blood meal taken by *Ae. aegypti* [33]. Another *Ae. aegypti* salivary protein acting on neutrophils and inflammation is Neutrophil Stimulating Factor 1 (NeSt1) [32]. Passive immunization against NeSt1 decreases pro-inflammatory cytokines such as interleukin-1β and CXCL2 and prevents macrophages from infiltrating the blood feeding site, thereby decreasing ZIKV [32].

A recent study by Sun and collaborators (2020) [39] described an *Ae. aegypti* venom allergen-1 (*Aa*VA-1) found to activate autophagy in dendritic cells and monocytes, promoting DENV and ZIKV virus transmission. *Aa*VA-1 is specifically expressed in the salivary glands of female *Ae. Aegypti*. In the vertebrate host, AaVA-1 competes with a leucine-rich pentatricopeptide repeat (PPR)-containing protein (LRPPRC), which is an autophagy antagonist on mitochondria. The study also suggests that *Aa*VA-1 may play a regulatory role in other immune responses, and the knockdown of *Aa*VA-1 resulted in the greatest reduction in ZIKV and DENV [39], suggesting *Aa*Va-1 as a potential vaccine candidate.

Several other proteins in *Ae. aegypti* saliva are known to enhance virus replication. The CLIPA3 protein, with serine protease activity, has been shown to disrupt the extracellular matrix, enabling DENV dissemination in vivo [36]. A 34kDa salivary protein was found to inhibit type I interferon (IFN), inhibit antimicrobial peptide (AMP) expression, and increase DENV replication in human keratinocytes [43]. This protein is found in significant amounts in *Ae. aegypti* saliva, and studies show that it is immunogenic, inducing significant levels of antibodies correlated with the intensity of exposure to mosquito bites [44,45]. These salivary proteins may also be suitable vaccine candidates, but further investigation on the effect of specific antibodies against them is needed.

To date, the only mosquito salivary-based vaccine currently in phase 1 trial is the Anopheles gambiae saliva vaccine (AGS-v), a synthetic peptide-based vaccine composed of four peptides (32–44 amino acids in length) predicted to be T-cell epitopes of proteins contained in *An. gambiae* salivary glands, but conserved across *Anopheles*, *Aedes*, and *Culex* mosquitoes [40,46]. Immunized individuals showed a significant increase in vaccine-specific total IgG antibodies and IFN-γ. The study determined that AGS-v was well tolerated, and, when adjuvanted, immunogenic, suggesting that the vector-targeted vaccine administration in humans is safe and could be a viable option for the increasing burden of vector-borne disease [40,41].

Several studies suggest there are salivary proteins present in the saliva of vectors that have detrimental effects on pathogen survival. The D7 salivary protein family is widespread among blood-sucking dipterans and represents one of the most abundant groups of proteins in arthropod saliva [47,48]. D7 are known platelet aggregation inhibitors that bind biogenic amines and eicosanoids [49]. We recently identified a D7 Long (D7L) (AAEL006424) salivary protein from *Ae. aegypti* mosquitoes that were highly abundant in salivary fractions that inhibited DENV replication [37]. Our studies demonstrated that this D7L protein was able to physically bind DENV virions and inhibit infection in vitro and in vivo [37]. Our preliminary studies also suggested that IgG antibodies against this D7L protein may be present at significantly higher levels in people with an active DENV infection [50]. In accordance with these results, a recent study showed that immunization against a D7L salivary protein from *Culex* mosquitoes increases disease severity with WNV in mice infected through a mosquito bite [51]. It is possible that antibodies against specific D7 with antiviral properties enhance virus infection. However, in an in silico analysis by Sankar et al. (2017) [52], two B-cell and T-cell epitopes were identified from a D7L and D7 short form (D7S). They postulate that immunity against these proteins decreases the efficiency of the blood meal process and could lead to protection against arboviruses [52]. Another *Ae. aegypti* salivary protein with a potent antiviral effect is aegyptin, a salivary protein known to block collagen-induced platelet aggregation [38]. Mice inoculated with aegyptin show a significant decrease in DENV replication [38,53]. It is also possible that antibodies against the D7L and aegyptin may inhibit their antiviral effect and promote virus transmission to the vertebrate host [37,50]. Therefore, further studies are needed to test these assumptions in vivo and in vitro.

One important question to ask now is why are people within endemic areas producing immune responses against mosquito saliva still becoming infected by the pathogens? The answer mainly relies on previous studies that suggest there are differences in the protein content of saliva from infected versus uninfected mosquitoes [54,55]. Although people may be exposed to significantly higher numbers of uninfected bites, the response against each immunogenic salivary protein is not the same as discussed previously. Thus, more studies are needed to elucidate natural immune responses against salivary proteins of the major vectors in people who have been chronically exposed versus those with seasonal or temporary exposure and correlate those studies to foresee how saliva-based vaccines would protect against arboviruses in each population.

## 3. Tick Salivary Proteins and Pathogen Transmission

The study of salivary proteins to control viral disease is more advanced in mosquitoes than in ticks. However, several salivary proteins contained in tick saliva have been studied as potential candidates to avoid tick feeding on a host or to prevent a pathogen from establishing an infection (Table 3). Lyme disease is the most common vector-borne disease in North America and Europe and can lead to serious health complications [56,57]. Probably the most notorious saliva–pathogen interaction studied in ticks involves the *Ixodes scapularis* salivary protein 15 or Salp15, named after its 15-kDa calculated molecular mass, which has been related to the transmission of *Borrelia burgdorferi* s.s., the causative agent of Lyme disease [58]. Salp15 expression is increased in infected ticks during feeding, and it binds directly to the spirochetes through the Outer Surface Protein C (OspC), protecting the pathogen from antibody-mediated killing [58,59]. Salp15 also inhibits CD4+ T-cell activation by binding to the CD4 coreceptor of mammalian T-cells, thereby inhibiting receptor ligand-induced early cell signaling [57]. Recent studies suggest that antibodies against Salp15 protect mice from suffering Lyme disease [60]. Another salivary protein whose name is based on the calculated molecular mass and also secreted by *I. ricinus* is known as Salp25D, a glutathione peroxidase homolog acting as a potent antioxidant in tick saliva [61,62]. Salp25D is highly immunogenic and associated with a decrease in tick infestation in immunized mice [62].

Another protein in the saliva of *I. ricinus* is the Tick Salivary Lectin Pathway Inhibitor (TSLP), which prevents the complement system from killing the bacterium [63]. The other salivary protein is the Tick Histamine Release Factor (tHRF), which has been related to the blood-feeding of the vector and facilitates the transmission of *Borrelia* spp. to the host—this was proven from trials with mice immunized with recombinant tHRF proteins and an altered blood-feeding process was observed in ticks, showing another good candidate for a vaccine based on blocking transmission [64]. Pre-clinical studies with these proteins in mouse models suggest they may represent good candidates for vaccines to interrupt the transmission cycle of Lyme disease [63,65].

*Rhipicephalus microplus*, the cosmopolitan species known as the Asian blue tick, is associated with the transmission of *Babesia bigemina*, *Anaplasma marginale*, *Anaplasma platys*, and *Ehrlichia* spp. [66]. An anti-tick vaccine based on a constructed transcriptome from all stages of *R. microplus* salivary glands displayed four salivary proteins named Rm39, Rm180, Rm239, and Rm76 in their recombinant forms, which showed significant activity in silico and then were used in vaccine preparations to test in cattle if these proteins could induce immunity against *R. microplus* ticks feeding at all stages of development. The study shows that the four recombinant proteins induced significant antigen-specific IgG antibody responses, especially a small amount of specific IgG1 antibodies that recognized epitopes after continuous immunization episodes, leading to a decrease in tick infestation during the blood meal process after multiple expositions at tick feeding sites [67,68].

Parasitoses, such as babesiosis and theileriosis (also known as Piroplasmosis), are important diseases in agriculture, and several salivary proteins from tick vectors are currently under study to evaluate their potential to avoid pathogen transmission or reduce the number of arthropods feeding in the host, leading to a reduced risk of diseases. In this regard, a calcium-binding protein known as calreticulin, which is a multifunctional protein present in almost all animal cells, is secreted by ticks into their hosts [69]. This has been a point of interest for some researchers trying to determine if the secretion of calreticulin during the feeding process is linked to modulating the parasite-host interaction. Evidence suggests an important role of calreticulin in host immunosuppression and anti-hemostasis during the blood meal process [70]. Additionally, a study on calreticulin from *Haemaphysalis qinghaiensis*, the vector of *Babesia* spp., and *Theileria* spp. in the Asian continent, is named HqCRT. Sheep vaccinated with its recombinant version suggest that the protein is immunogenic and recognized by specific antibodies in the sheep serum and induces a significant increase in tick mortality after blood feeding [71]. Other calreticulin proteins from species affecting livestock are found in larvae and engorged female salivary gland extracts of *Haemaphysalis longicornis* (rHlCRT) and *R. microplus* (rBmCRT) [72].

Tick-borne encephalitis virus (TBEV) is the most important vector-borne virus infection in Europe and Northern Asia [73]. The major vectors of TBEV are *I. ricinus* (associated with the European TBEV subtype) and *I. persulcatus* (associated with the Northern Asia TBEV subtypes) ticks [74]. Cement proteins are secreted by ticks to attach the mouthparts to the host during blood feeding. A 15kDa protein called a 64-tachykinin-related peptide (64TRP) was identified as a cement protein in *Rhipicephalus appendiculatus* ticks, increasing the transmission of TBEV. Another study suggests that antibodies against this protein confer protection against TBEV transmitted by *I. ricinus* in a mouse model [75]. Some studies suggested that vaccination with 64TRP increased antibody titers and induced the infiltration of white blood cells in immunized mice [75,76]. The 64TRP was also found to increase CD4^+^ and CD8^+^ T lymphocyte, thereby conferring antiviral protection and delayed hypersensitivity response [75]. In hamster, guinea pig, and rabbit models, 64TRP may present a dual-action against TBEV by impairing attachment and feeding at feeding sites and cross-reacting with the midgut antigens, resulting in the early mortality of engorged *I. ricinus* ticks after feeding [75,76].

**Table 3:** List of salivary proteins studied as potential vaccine candidates.

Pathogen	Protein	*Species*	Function	Phase	References
Tick Borne Encephalitis virus	64TRP	*Rhipicephalus appendiculatus*	Disrupts the skin feeding site and then specific anti-64TRP antibodies cross-react with midgut antigenic epitopes.	Pre-clinic	[75,76]
Lyme disease	SALP15	*Ixodes scapularis*	Inhibition of CD4+ T-cell activation by binding to the CD4 coreceptor of host T-cells, inhibiting receptor ligand-induced early cell signaling.	Pre-clinic	[58,77]
Lyme disease	SALP25D	*Ixodes scapularis*	Detoxified reactive oxygen species at the tick–bacteria–host interface that provides a survival advantage to B. burgdorferi.	Pre-clinic	[62,64]
Lyme disease	TSLP	*Ixodes scapularis*	Protects B. burgdorferi from the complement system.	Pre-clinic	[78]
Lyme disease	tHRF	*Ixodes scapularis*	Facilitates the transmission of Borrelia spp. to the mammalian host.	Pre-clinic	[64]
Babesiosis and Theileriosis	HqCRT	*Haemaphysalis qinghaiensis*	Induces host good humoral response against ticks feeding process.	Pre-clinic	[71,79]
Babesiosis and Theileriosis	HlCRT	*Haemaphysalis longicornis*	Induces host good humoral response against ticks salivary extracts.	Pre-clinic	[72]
Babesiosis and Theileriosis	rBmCRT	*Rhipicephalus microplus*	Induces host good humoral response against ticks salivary extracts.	Pre-clinic	[72]
Anaplasmosis	Sialostatin	*Ixodes scapularis*	Affects the formation of inflammasomes promoting host infection	.Pre-clinic	[80,81]

## 4. Natural Antibody Responses against Arthropod Salivary Proteins and Disease

The design and implementation of proper tools for the evaluation of vaccines’ efficacy need to be set in place when designing the vaccines. These tools are needed to accurately measure the exposure to specific disease vectors and to calculate disease risks to guide public health policy after the implementation of new or improved vaccines against vector-borne diseases. We believe it is important to determine mosquito feeding intensity through measuring IgG antibodies against specific salivary proteins as a means of determining how much exposure to mosquito bites a person has suffered after vaccination.

A significant number of salivary proteins are immunogenic and induce antibody responses that correlate with the intensity of exposure to mosquito bites [82,83]. These antibody responses are mainly the IgG type, with the IgG4 subclass being the most prominent [84,85,86]. Significantly higher levels of IgE antibodies are present in people with allergic reactions against arthropod saliva [85,86]. Studies indicate that saliva from both mosquitoes and ticks contain immunogenic proteins [83,87,88]. Our study using *A. americanum* SGE showed significant changes in antibody levels between seasons. Specifically, we observed a significant decrease in IgG antibodies in the fall compared to those shown by the same volunteers in the summer [87]. IgM antibodies are also elicited against these salivary proteins and our studies showed that IgM antibodies against whole salivary gland extract (SGE) correlated with IgG antibody levels against DENV [88]. However, they have a low correlation with bite exposure intensity or risk of suffering a disease [89,90].

Antibodies against an arthropod’s saliva are short-lived [40,87,89], diverse, and not equally produced against all salivary proteins [91]. Thus, only a few individual proteins have been evaluated as markers for vector-bite exposure [91,92]. As previously discussed, the 34kDa protein is highly immunogenic and there is currently one *Ae. aegypti* peptide derived from this protein, the Nterm-34kDa, that has been evaluated as a proxy to quantify exposure to *Aedes* bites (Table 4). The level of IgG antibodies against the Nterm-34kDa is positively correlated with the intensity of exposure [44,45], but the correlation with disease status or active arbovirus infection is under study. The salivary protein 34k2 from *Ae. albopictus* has also presented a significant correlation with exposure to mosquito bites [93].

Interestingly, antibody responses against arthropod salivary proteins may vary according to factors such as age or seasonality [50,87,89]. Several studies suggest that the IgG responses to mosquito salivary proteins may serve as surrogate biomarkers for exposure to mosquito bites and as an indirect marker for disease risk in travelers and individuals living in endemic areas, since IgG antibodies decrease significantly in the absence or in the event of a decrease in exposure to mosquito bites after vector control interventions [88,90]. Other studies have also shown that people living in houses where *Ae. aegypti* larvae are found present significantly higher IgG antibody levels against the SGE of this mosquito species than people whose houses are mosquito-free. Further studies later suggested that the level of IgG antibodies against salivary proteins are also correlated with socioeconomic status and the presence of disease, possibly because mosquito presence in households could be associated with factors such as access to running water, water storage, and access to public waste systems [94].

## 5. Conclusions

**Figure 2:**
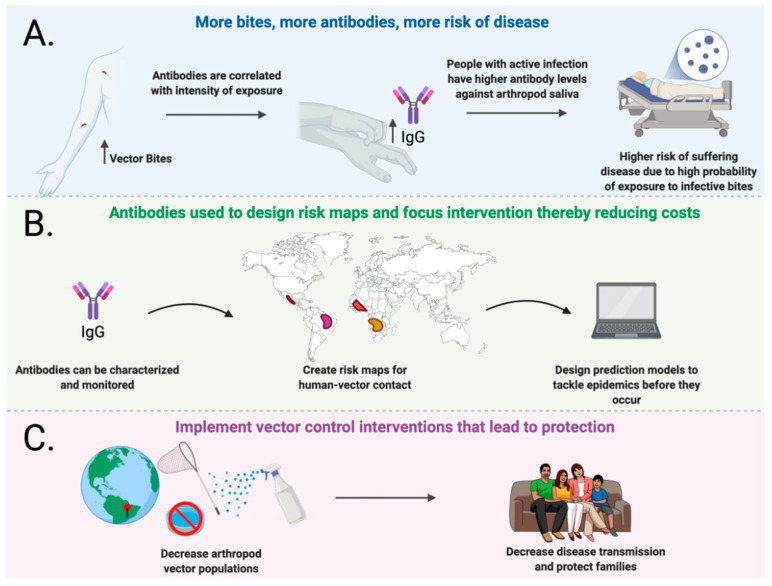
Use of antibodies against salivary proteins for epidemiological purposes. (**A**) Higher antibodies against specific vector salivary proteins are found in people exposed to bites and presenting disease. (**B**) These antibody levels can be used to design risk maps and to identify vector-human-contact “hot spots” within a community. With the proper model, changes in antibody levels can be used to predict epidemics before they occur. (**C**) The identification of “hot spots” may reduce intervention cost by directing efforts to areas where more human contact rates are observed protecting the entire community (figure created with BioRender.com and MindtheGraph.com) (accessed on 4 March 2021).

Salivary proteins from the main arthropod vector of human pathogens may represent viable alternatives to increase the efficacy of vaccine candidates against vector-borne diseases. Some of these proteins may even represent an alternative to tackle several pathogens sharing the same vector or vectors within the same family or subfamily. Other salivary proteins can also be used as biomarkers of exposure, which is useful in measuring the efficacy of salivary-based vaccine candidates by allowing the direct measurement of exposure to vector bites and the risk of disease in vaccinated versus unvaccinated populations. The therapeutic efficacy and potential of salivary proteins has created a new scientific development that can be used in vaccine development to tackle viruses and vector-related diseases affecting humans and their environment (Figure 2).

Studies using multi-disciplinary approaches, such as the use of bioinformatic analysis for epitope prediction, protein folding, and post-translational modifications on a vaccine candidate molecule, could strengthen the development of anti-vector vaccines. In addition, the development of multivalent vaccine formulations consisting of vaccines targeting both vectors and pathogens would provide a significant leap within this research area to propel it forward from bench to bedside.

## Figures and Tables

**Table 2:** List of salivary proteins studied as potential vaccine candidates.

Pathogen	Protein	Species	Function	Phase	References
Zika virus	NeST1	*Aedes aegypti*	Prevents early changes in inflammatory milieu.	Pre-clinic	[32]
Zika virus	AgBR1	*Aedes aegypti*	Prevents early changes in inflammatory milieu.	Pre-clinic	[33,34]
Zika virus	LTRIN	*Aedes aegypti*	Binds and inhibits the lymphotoxin-β receptor (LTβR)	Pre-clinic	[35]
Dengue virus	CLIPA3	*Aedes aegypti*	Disrupts extracellular matrix allowing virus dissemination	Pre-clinic	[36]
Dengue virus	D7	*Aedes aegypti*	Inhibits DENV infection in vitro and in vivo.	Pre-clinic	[37]
Dengue virus	Aegyptin	*Aedes aegypti*	Blocks collagen-induced platelet aggregation	Pre-clinic	[38]
Dengue and Zika virus	*Aa*VA-1	*Aedes aegypti*	Increases viral replication in macrophages and dendritic cells.	Pre-clinic	[39]
Mosquito transmitted diseases	AGS-v	*Anopheles gambiae*	Increases vaccine-specific IgG antibodies and cellular responses	Phase 1	[40]

**Table 4:** Natural antibody responses against arthropod salivary proteins.

Spp.	Salivary Protein	Antibody Response	References
*Aedes aegypti*	34kDa	IgG	[92]
*Aedes albopictus*	34k2	IgG	[93]
*Aedes communis*	36kd	IgE, IgG4	[85,86]
*Aedes aegypti*	D7	IgG	[50]
*Aedes caspius*	SGE	IgG	[83]
*Aedes aegypti*	SGE	IgG, IgM	[84,88]

## Data Availability

Not applicable.
